# Interactive Course Design and Development for Cognitively Inspired Distance International Chinese Education

**DOI:** 10.1155/2022/5040920

**Published:** 2022-10-13

**Authors:** Qi Zhang

**Affiliations:** School of Foreign Languages, Beijing Institute of Technology, Beijing 100081, China

## Abstract

This paper conducts in-depth research and analysis on the design and development of interactive courses for distance international Chinese language education using the cognitive heuristic model. The A-frame framework and JavaScript (JS) language are used to complete the construction of the virtual supermarket environment and initially design the basic framework of task-based courseware for Chinese language teaching based on virtual reality technology. Experts were invited to conduct two rounds of prestudy on the index system and determine the evaluation indexes. The international students in the junior Chinese class of X University were randomly selected as the research subjects and divided into experimental and control groups for the experimental intervention teaching. The content and form of classroom teaching were the same for both groups, and they were asked to review and consolidate the grammar points learned in class after class. The only intervention was that the experimental group was required to conduct microlearning after class with the classroom grammar points, while the control group was not required to do the same form of review after class. In addition, the students in the experimental group were followed up as a case study, and their attitudes, behaviors, and knowledge proficiency in the microlearning were recorded in detail to assess the students' acceptance and satisfaction of the microlearning from the perspective of qualitative analysis. It was found that the microlessons for teaching and learning Chinese as a foreign language can stimulate learners' interest in learning, achieve good interactive effects, and contribute to the improvement of learners' performance. This paper combines virtual reality technology, fully explores the role that immersive VR technology can play in providing cultural scenarios in the target language, and maximizes the use of existing technological vehicles and resources for Chinese teaching application design, to solve the challenges of poor interactivity in online teaching and the lack of socio-cultural contexts that have long existed in overseas Chinese teaching.

## 1. Introduction

Under the background of “One Belt, One Road,” with the increasing comprehensive national power of China, more countries are conducting trade and economic exchanges with China, the number of people learning Chinese is increasing day by day, and the “Chinese language fever” has developed to a new height. The scope and scale of foreign economic and cultural exchanges are also expanding rapidly, and an increased number of foreigners are choosing to learn Chinese, learn about Chinese culture, or go to China to engage in economic activities and business cooperation [1]. Chinese culture, as the main representative of “soft power,” has also been included in the fight and containment, and the development of offline Chinese international education entities has been facing serious challenges. 2020 saw the sudden outbreak of the new crown pneumonia epidemic in China and its rapid spread around the world. To “protect themselves,” many countries have also introduced border closures, restrictions on entry and exit, etc. Whether due to economic costs or the risk of spreading the epidemic, the willingness of foreign Chinese language learners to come to China to learn Chinese has been restricted, and the number of foreign students coming to China has dropped sharply [2]. Even overseas Confucius Institutes have seen a decline in the number of students. As a result of the epidemic, international Chinese language education has been forced to shift from offline teaching to full online teaching, thus exposing many problems such as poor interactivity, lack of presence required for language teaching, scarcity of shared teaching resources, and lack of attractiveness to younger students.

Distance learning is commonly known as online teaching. Online teaching is learning on the Internet, where teachers and students use computers or other electronic devices such as cell phones to complete classes, exercises, exams, and other teaching sessions. The characteristics of online teaching are three: first, it breaks the constraints of time and space; second, it can make full use of the advantages of multimedia; third, it can meet the diversified needs of students and personalized learning. However, the lack of classroom learning environment and atmosphere, the lack of learner interaction, and the low interactivity of teacher-student communication in online teaching have brought troubles to both learners and teachers. Especially for Chinese language teaching, since it is a language course, it needs to be more interactive than theoretical and knowledge-based courses [3]. In the Chinese classroom environment, teacher-student interaction and learner interaction are indispensable, and the interactive situation is more realistic and easier for teachers and students to empathize in a real environment. Therefore, how to enhance the effect of interactive teaching in online Chinese teaching is an urgent problem to be solved in online Chinese teaching. The Education Informatization 2.0 Action Plan officially announces that education informatization has entered the 2.0 era from 1.0. Digital technology with artificial intelligence as the core, integrating big data, cloud computing, virtual reality, 5G, Internet, and other technologies, is becoming an important force to support the transformation of education and promote the transmutation of education to the form of intelligent education. In the past, due to the limitation of technology, we could not break through the limitation of time and space to get the learning experience in a real situation at will, but now, virtual reality technology can make this “luxury” become reality [4]. As Virtual Reality (VR) becomes a more pervasive information medium, the user experience will continue to evolve along with the technology. VR is not just the 3D version of the 2D video, it is a new way of consuming media that brings new visuals and experiences to the user. With this comes a constant evolution of educational concepts, methods, and technologies. As a new type of teaching media technology, virtual reality (VR) technology has obvious advantages in sensory stimulation, scene construction, and human-computer interaction, which enriches the contextualized teaching methods of Chinese teaching and provides technical support for Chinese teaching. As we all know, “virtual reality” has become one of the emerging technologies in the 21st century. In the past two years, the application of virtual reality technology in education has become increasingly extensive, providing more technical support for education. The live broadcast session in the class is mainly for real-time interaction, and the types of interaction include situational interaction, game-based interaction, and topic-based interaction. In the after-school session, there are real-time interaction using instant messaging tools and delayed interaction using online learning platforms. The types of interaction mainly include answering questions and discussions.

In the era of digital devices, we can achieve better learning through technology. VR seems to be the next step in the development of education, where learners can learn language knowledge anytime and anywhere according to their needs to improve their language skills, which will be an inevitable trend in the future development of education. With the rapid development of science and technology, 5G technology is about to enter our life. 5G network technology has greatly improved the bandwidth, and the high speed and low latency make the transmission speed of image and audio get a qualitative leap [5]. This has also driven the development of the virtual simulation field, and technology applied to the virtual simulation field so that the virtual simulation technology interface is clearer, smoother, more responsive, and more comfortable to operate. Such a development trend also further triggered people's attention to the virtual simulation learning platform on mobile, and many people invested in the research in this field [6]. The popularity and development of Internet technology have had a profound impact on Chinese language teaching and have also promoted changes and innovations in the form of Chinese language teaching. Especially after the outbreak of the new Chinese language epidemic, online teaching has highlighted its advantages across time and space. However, because most teaching units are passive and “rushed,” online Chinese teaching has also revealed many problems that seriously affect the effectiveness of online teaching, one of the most prominent ones being classroom interaction. In this paper, we use the cognitive heuristic model to study the design and development of interactive courses for distance international Chinese education and build a virtual simulation mobile learning platform for the international Chinese language, so that learners can experience the profundity of Chinese language in a real environment and improve their Chinese oral communication skills.

## 2. Related Works

The development of network technology has greatly contributed to the reform of traditional education, and the concept, means, and content of education has undergone great changes. Distance education, as an educational carrier of modern technology, is getting increased attention from academic circles. With the development of economic construction and the innovation of science and technology, distance education is developing rapidly [7]. However, many problems have emerged with it, and the trend of marketization of distance education is obvious. Under the modern market competition environment, how to promote the development of distance education and occupy a place in the international distance education market through management has become a research topic for scholars. Rogers et al. pointed out that the original purpose of distance education is to meet the actual needs of students, and they showed in the related discussion that the roles and roles of both teachers, as instructors, and students, as recipients of education, are crucial in students' independent learning [8]. Distance education provides sufficient resources for students to learn independently, while students should give full play to their subjective initiative under the guidance of teachers, select and use these resources independently, set reasonable learning programs according to their proficiency and knowledge accumulation, and carry out learning in a planned manner. This is the main content of the theory of self-directed learning in distance education. Ouyang and Stanley combined the connotation characteristics of distance education and the basic knowledge of engineering supervision to summarize and conclude the curriculum design of distance education, proving that after the introduction of project management theory, it is possible to develop distance education curriculum resources more easily and more quickly [9]. Wang et al. developed China's distance education theory comprehensively and systematically based on their advanced experience in foreign distance education [10]. He introduced the distance education theory that deals with distance education itself and distance education learning and the driving forces of both. He also pioneered China's distance education development strategies, distance education technologies, indicators for distance education assessment, and even pioneered the economics of distance education in conjunction with the field of economics. The *p*-value of the independent samples *t*-test of the pre-test scores of the experimental group and the control group was greater than 0.05, indicating that there was no significant difference. From this, we can know that there is no significant difference in Chinese proficiency between the experimental group and the control group at the beginning of the controlled teaching experiment, that is, the experimental subjects conform to the principle of experimental homogeneity.

Currently, virtual reality technology is more widely used and is present in several disciplines and different fields. As we enter the second decade of the 21st century, there is an urgent need to move away from traditional learning methods. With the rapid shift to virtual reality in the field of teaching technology, many educational research institutions at home and abroad have innovated educational methods learning methods and launched exploratory research on VR education and have achieved many research results with practical application and reference value to provide a reference for subsequent research on the application of virtual reality in education. Gong et al. proposed a vision based on the application of VR technology in Chinese language teaching, i.e., using VR technology to create a virtual classroom and social language learning environment [11]. The key to the application of VR technology in language teaching is the design of teaching activities and learning tasks, which provides a clear direction for this paper's conception of Chinese language teaching based on existing VR social games. This provides a clear research direction for the idea of teaching Chinese based on existing VR social games. Songco et al. put forward the assertion that virtual reality technology is needed for teaching Chinese as a foreign language and believe that virtual reality technology should be applied to teaching Chinese as a foreign language to create a daily speaking acquisition environment [12]. He was the first to envision the “combination of classroom teaching and natural acquisition” model of teaching Chinese as a foreign language and explained the key points and conditions for the implementation of this teaching model. Although the technical conditions at the time were not certain, Klaproth et al. research philosophy was very forward-looking, as he said, “if we wait until the technical conditions are fully developed before starting to study these topics, we will be behind the times in teaching” [13]. Klaproth et al. consider the impact of physical activity on the quality of teaching and learning in their instructional design, concluding that the use of physical activity has implications for a better understanding of more abstract knowledge. The effects of the body on cognition are present at all stages of cognitive development, with the body's influence on cognition being timely and immediate when learning simple content, and deeper and less perceptible for understanding complex abstract knowledge. This means that the embodiment of the teacher's behavior during instruction can have an impact on student acquisition, concerning the effective input of the classroom environment [14]. The results of the experiment set the stage for later applications of language acquisition and embodied cognition in second language acquisition. All values fall between 0.586 and 0.936, which are far greater than 0.16, and the effect of this factor extraction is acceptable.

At present, distance international Chinese teaching is booming, but there are certain controversies and problems in the theory and practice of distance teaching, and the most prominent one is that its quality assurance system is not perfect and sound, so it is especially necessary to establish a complete system of distance international Chinese teaching. To this end, it is necessary to analyze the characteristics of the cognitive model of distance international Chinese teaching from the four dimensions of cognitive coding, information perception, contextual cognition, and metacognition in the cognitive theory system, and establish a new model of distance international Chinese teaching based on cognitive linguistics and cognitive psychology theories. In addition, we propose the establishment of a curriculum resource base, a hypertext and hypermedia teaching resource base, and a basic database of Chinese characters.

## 3. A New Model of Distance International Chinese Teaching Based on Situational Cognitive Theory

Distance international Chinese language teaching is based on computer software, which is used to spread Chinese culture and teach the Chinese language through the Internet. Unlike group teaching, it has no class organization and is completely outside the classroom; it is also different from individual teaching, where there is no face-to-face teaching by the teacher and the learning individual operates and learns completely independently. With the development of modern information technology, distance international Chinese teaching supported by information processing theory and cognitive science theory has great potential for optimizing the Chinese learning environment and promoting the spread of the Chinese language and Chinese culture [15]. At present, distance international Chinese teaching based on information processing theory and cognitive science theory has moved from a single form to a comprehensive one, and it has become increasingly mature and diversified. Distance international Chinese teaching has changed the teacher-student relationship in the original classroom teaching and has created a new way of human-computer interaction. Learners log in through the website, click on the pages, display the learning contents on the screen, and follow the learning procedure to memorize, practice, and remember step by step. Learners establish virtual teacher-student and classmate relationships through the computer, and learners raise their hands to speak and cooperate through the Internet. The characteristics of distance international Chinese teaching are based on computer information technology; therefore, it is necessary to explore the human-computer relationship and cognitive activities.

In the field of design, context refers to the scene or environment where user behavior occurs, and in some design studies, context is also considered to be the coordinated relationship between the human-computer-environment. Context refers to the teaching atmosphere felt by students in the learning process, including the physical environment created by various hardware and software facilities and includes the teacher's charm, inspiration, and interactivity, which is the learning behavior of students. It is the scene and environment in which the students perform their learning behaviors. In general, context is the element that is relevant when an individual interacts with the environment [16]. The role of context in learning is to help learners construct the meaning of learning, to establish the relevance of learners' cultural background and life experiences to knowledge, to make knowledge more vivid, and to improve the learning effect and experience. In the basic research and practice of contextual cognitive theory, the contextual nature of cognition and learning is pointed out, and the focus of research shifts from individual cognition to the relationship between individuals and social situations and people's participation and activities in such situations. The main views include the contextual view of knowledge and the contextual view of learning. The contextual view of knowledge believes that the nature of knowledge is contextual. The contextual view of learning emphasizes the importance of the community of practice. The contextual cognitive theory believes that learning is rooted in context and that learning is legitimate marginal participation in a community of practice.

**Figure 1:**
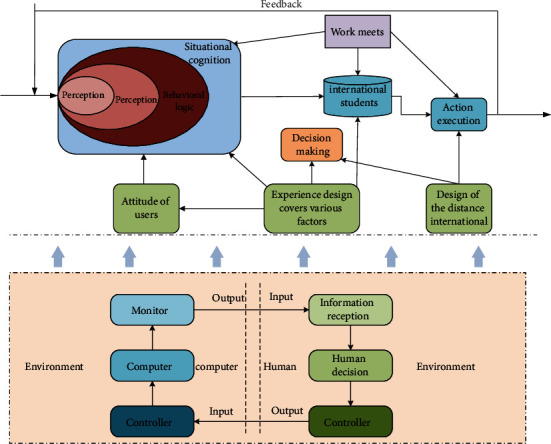
Human-computer interaction model of user experience design based on contextual cognition.

User experience is the emotion and attitude of users when they use a specific product, system, or service. Context-based user experience design covers various factors in the interaction process of humans, products, and the environment, and the intervention of context has a comprehensive impact on user experience to the extent that it is thus seen that user experience is closely related to context. As shown in Figure 1, the human-computer interaction model of UX design based on contextual cognition is shown. The contextual cognitive theory emphasizes learner-centeredness, so the design of the distance international Chinese teaching model should pay attention to the individual differences of international students and provide personalized Chinese learning services. In the application of the personalized learning concept, there are still shortcomings such as incomplete user information collected, isolated user learning data, and lack of correlation. Ignore the systemic and logical nature of language learning itself, and lack of concentration and immersion in learning; compared with offline classroom learning, online Chinese learning is mainly self-study, lacking opportunities to communicate and interact with other learners and teachers, and lack of language Opportunities for practice and practice are not conducive to cultivating teacher-student relationship. The design of the remote international Chinese teaching model requires the multi-dimensional acquisition of international students' user data to help shape an accurate three-dimensional user portrait of international students, predict their online Chinese learning behaviors and tendencies, and recommend more targeted Chinese learning content to international students.

From the perspective of the learning environment, the diverse learning scenarios for learners in the Internet learning environment bring convenience to learners while the distracting factors of learning are also increasing, and learning noise is high. While learners' online learning lacks self-control and effective face-to-face supervision, their learning behavior is not sustainable and their learning stickiness is low; the fragmented learning characteristics under mobile learning also neglect the systematic and logical nature of language learning itself, and their learning concentration and immersion are insufficient. Compared with offline classroom learning, online Chinese learning is mainly self-learning, lacking opportunities to communicate and interact with other learners and teachers, and lacking language practice and compared with offline classroom learning, online Chinese learning is mainly self-learning, lacking opportunities to communicate and interact with other learners and teachers, and lacking opportunities for language practice and practice, which is not conducive to cultivating teacher-student relationship.

One of the most important dimensions of the quality characteristics of the scale is the reliability test, which is broadly defined as the degree of stability and reliability of the scale, and narrowly defined as the degree of consistency in repeated measurement situations, guaranteeing the exact and stable data when the scale is used. The Cronbach's alpha coefficient (Cronbach's alpha) is a commonly used reliability test. The Cronbach's alpha coefficient ranges from 0 to 1, with alpha ≥0.9 indicating very good reliability, 0.8 ≤ alpha <0.9 indicating good reliability, 0.7 ≤ alpha <0.8 indicating high reliability, 0.6 ≤ alpha <0.7 indicating fair reliability, and 0.5 ≤ alpha <0.7 indicating good reliability. 0.5 ≤ *α* < 0.6 indicates acceptable and additional or modified questions, and *α* < 0.5 is not acceptable. The overall internal consistency coefficient of this scale was obtained through SPSS software analysis of the data of the teaching evaluation scale in the view of this cognitive theory. There are 22 indicators for validity analysis in this evaluation scale, that is, the reliability analysis was conducted for 22 questions except for the basic information, and the reliability coefficient was 0.858, which is greater than 0.8, indicating that this reliability is ideal.

**Figure 2:**
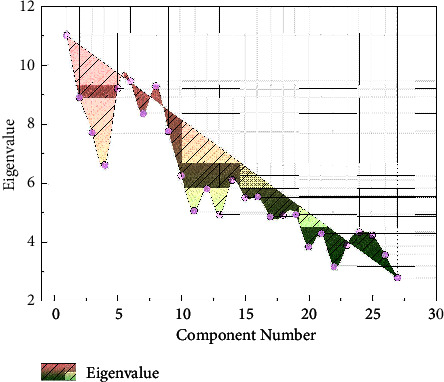
Factor diagram of prestudy results.

Analysis of the data of teaching evaluation scale in the perspective of this cognitive theory by SPSS software. The KMO value was 0.734, which was suitable for factor analysis according to the criteria given by Kaiser [17]. Bartlett's spherical test gave a companion probability of 0.0001, which was less than the significance level of 0.05, so the null hypothesis is rejected and the correlation coefficient matrix is considered significantly different from the unit array and the original variables are suitable for factor analysis. Analyzing the commonality data of all variables, i.e., the common factor variance, all values fall between 0.586 and 0.936, which are much greater than 0.16, and the effect of this factor extraction is acceptable. From the data reflecting the image matrix, the sampling fitness number of each item is greater than 0.5, there are no items that need to be deleted, and the variables can all enter the factor model. The results of factor extraction and factor rotation from the data after doing factor analysis are shown in Figure 2. Four common factors were extracted to describe 79.798% of the variance of the original variables, and overall, the information of the original variables was not much lost, and the extracted factors were enough to reflect most of the information of the original variables. Combined with the various functions of the HTML5 online editing platform, fragmentation and learning anytime, anywhere can be achieved. The mobile learning resources for spoken Chinese are mainly divided into three parts: word learning, communication practice, and sharing feedback.

## 4. Virtual Reality and Distance International Chinese Education Interactive Course Design Ideas

This learning software is mainly based on the international Chinese textbook and selects different training scenarios according to the needs of students of different levels. It is mainly divided into three level modules: Elementary International Chinese, Intermediate International Chinese, and Advanced International Chinese. Students are familiar with the text offline, and the teacher teaches and explains the knowledge points offline and conducts basic knowledge point drills. In the second stage, students enter the online Chinese virtual simulation training, which has two options. Students can operate on the PC side and put on virtual simulation VR glasses in a specific situation or laboratory, which makes the simulation more realistic and richer in experience. Another option is to choose the cell phone mobile end of the exercise, the mobile platform end of the device can be used with the corresponding cardboard glasses [18]. You can also choose to directly enter the software application platform, do not wear glasses, the view is a 3D stereoscopic animation effect, and students can also be in the corresponding scenarios for role simulation, to achieve good learning results. To operate, you only need to download the software from the mobile device store, enter the installation process, and use it after success. Each user needs to have his or her nickname to log in to the platform, and can fill in his or her real name for a stronger sense of immersion in the scenario experience. Students' motivation in online learning can be affected by the transfer of learning places. Due to the lack of such a real classroom environment for online teaching, students are alone in their own learning space, and some classroom activities that can be completed in offline classrooms cannot be carried out online.

**Figure 3:**
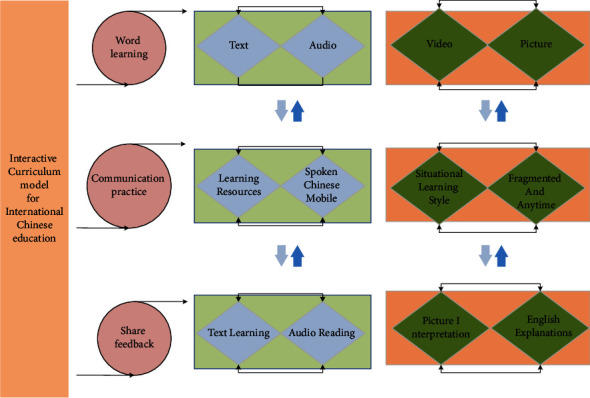
International Chinese education interactive course model.

Different mobile learning resources will be designed for different subjects and different users. The spoken Chinese mobile learning resources will be designed in strict accordance with the characteristics of spoken Chinese learning, emphasizing the immersion and situational learning style. Therefore, the Spoken Chinese mobile learning resource is a teaching resource that classifies film and TV resources into topics and levels of difficulty and works with various functions of the HTML5 online editing platform to realize fragmented and anytime, anywhere learning [19]. The mobile learning resource for spoken Chinese is divided into three parts: word learning, communicative practice, and feedback sharing. Among them, word learning includes text learning, audio reading, and picture interpretation. The text content includes English explanations and pinyin, in addition to the display of example words and sentences. Different texts are differentiated by adopting different fonts and sizes. It is easier to learn words by putting them into a context or situation. The model of the international Chinese education interactive course is shown in Figure 3.

A-frame is a web framework for creating virtual reality experiences. It uses the easy-to-get-started HTML language, but it includes not only the simple tag-based language HTML, and simple to create a 3D environment. At the core of the A-frame is a powerful entity-component-system (ECS) framework. It provides a declarable, extensible, programmable structure for the “three.js” framework. The HTML language is easy to read and understand, and easy to copy and paste. It is also possible to access JavaScript (JS) scripts, DOM APIs, Three.js, VR, and WebGL for in-depth editing and design. To develop A-frame, simply edit an HTML file and add a line of code to the header of the file to reference the A-frame framework JS script file. Once the code is edited, the file can be opened with a browser to get a VR interactive scene. A-frame provides a series of tag elements that can be used directly such as <a-box>, <a-sky>, called Primitives, which are already made “entity-component” wrappers. “The surface abstraction layer of a-frame is simple HTML and DOM, but deeper down it is an entity-component framework based on three.js. The number of international students studying in China has decreased sharply, and even overseas Confucius Institutes cannot escape the dilemma of the decrease in the number of students studying in China.

Since HTML and JavaScript languages can be played in the browser, the use of A-frame generally does not require installation. Some sites offer online code editing to deploy and host web code in real-time, such as glitch and code pen. For example, glitch and code pen, but if you choose not to develop your project online, then when you open the HTML file directly in your browser, the images in your project will not be rendered due to the browser permissions of the file protocol, and you will need to set up a local server to use [20]. The most convenient way to do this is to use the terminal to run commands under the path where the HTML file is located. Then, the terminal will prompt for the local URL and port of the server, which can be copied to the browser to display the HTML project file normally.

The teaching of physical culture focuses on giving learners the most realistic and natural social and cultural situations, so both the scenario design and the script design must be based on the principle of authenticity. Only by designing the scenarios most realistically will learners be able to communicate with Chinese people in the future without feeling that the content of the physical performance is too different from the actual application. Of course, to achieve the most realistic social and cultural context, it is most important to invite native Chinese speakers to participate in the exercises so that learners can experience social interaction with native Chinese speakers. The learning content and the learning goal of physical culture teaching are mainly about the learners' ability to use the target language appropriately and comfortably to interact with people in the target language in a specific context, so we must consider the interactivity in the design process, both to give the learners an immersion experience and to use Chinese appropriately [21]. The interaction with the teacher, with peers, with native Chinese speakers, and with the scenario during the physical performance process must be considered. The learning content and the learning goal of physical culture teaching are mainly about learners' ability to use the target language appropriately and comfortably to interact with people in the target language in a specific context. The interaction with the teacher, with peers, with native Chinese speakers, and with the scene during the physical performance.

## 5. Practice and Evaluation of Interactive Courses in Distance Chinese Education

Due to the epidemic, the number of international students coming to China to study Chinese was limited, and most universities also adopted strict entry and exit management measures, and no two eligible classes were found. Therefore, the study decided to select students from the junior Chinese language class in the School of International Education at X University as the study subjects and randomly divided them into experimental and control groups, using their pseudonyms to protect the privacy of the subject students. A total of 11 Chinese language learners from 8 countries, including the UK, Russia, Ghana, South Africa, Morocco, Nigeria, Zimbabwe, and Bangladesh, participated in the experiment and were able to communicate in English. Through the preliminary interviews and questionnaire tests, the author learned that the Chinese proficiency of these 11 students varied widely [22]. Although all of them were newly arrived international students in China, some of them had studied Chinese for two or three years before, and some had only six months of Chinese foundation. There were 6 students in the experimental group and 5 students in the control group. The results of the independent sample *t*-test of the pretest scores of the two groups were indicated. The data showed that the *p*-value of the independent sample *t*-test for the pretest scores of the experimental group and the control group was greater than 0.05, which means there was no significant difference. Thus, we can know that the Chinese language proficiency of the experimental group and the control group did not differ significantly at the beginning of the controlled teaching experiment, i.e., the experimental subjects conformed to the principle of experimental homogeneity. It is also different from individual teaching, there is no teacher face-to-face teaching, and the learning individual operates and learns completely independently. With the development of modern information technology, distance international Chinese teaching is under the support of information processing theory and cognitive science theory.

**Figure 4:**
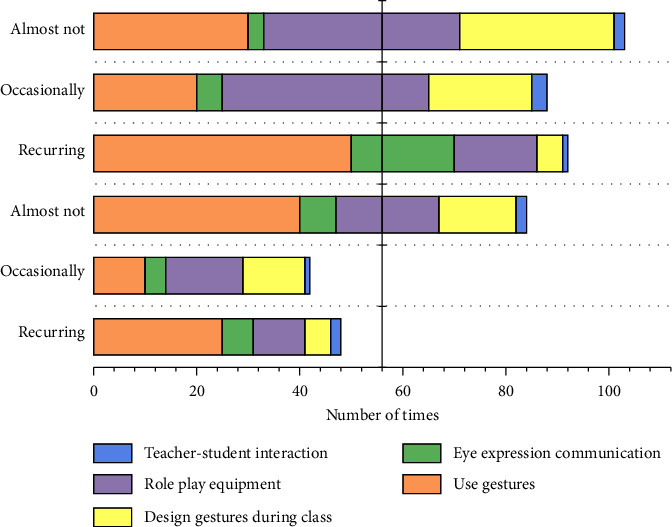
Statistical results of teachers' body movements and expressions of participation.

Some questions about the use of body language in classroom instruction. For example, when teachers were asked whether they consider or intentionally design instructional gestures when preparing for class, only 16.03% of teachers chose to consider this aspect and teach gestures that are carefully designed and have a pedagogical purpose, while 12.21% of teachers hardly consider the use of gestures. The frequency with which teachers organized students' use of physical involvement activities in the classroom also indicates that most classroom instructional sessions in which students use physical involvement activities are still relatively rare. Teachers' intentional or unintentional use of body language in the classroom and insufficient attention to the instructional design of body movements should also draw teachers' attention to the physical activities and reasonable use of students' body activities to promote students' cognitive development. As shown in Figure 4, most teachers would often use gestures to participate in teaching activities, 47.33% of teachers would often use eyes and expressions to communicate with students, and there were almost no cases of never using gestures and eye expressions in the classroom. This indicates that it is very common for teachers' body movements and expressions to participate in teaching and learning in the classroom environment.

The physical gestures can achieve a better teaching effect on the one hand and get more recognition from the students on the other hand, and the cooperation in the class will be improved. Therefore, I will prepare for the class by designing the teaching sessions, such as how to match the gestures with the vocabulary teaching, and designing some games to support the teaching. What follows is the continuous update and development of educational concepts, methods, and technologies. As a new type of teaching media technology, VR technology has obvious advantages in sensory simulation, scene construction, and human-computer interaction. It enriches the situational teaching methods of Chinese teaching and provides technical support for Chinese teaching.

**Figure 5:**
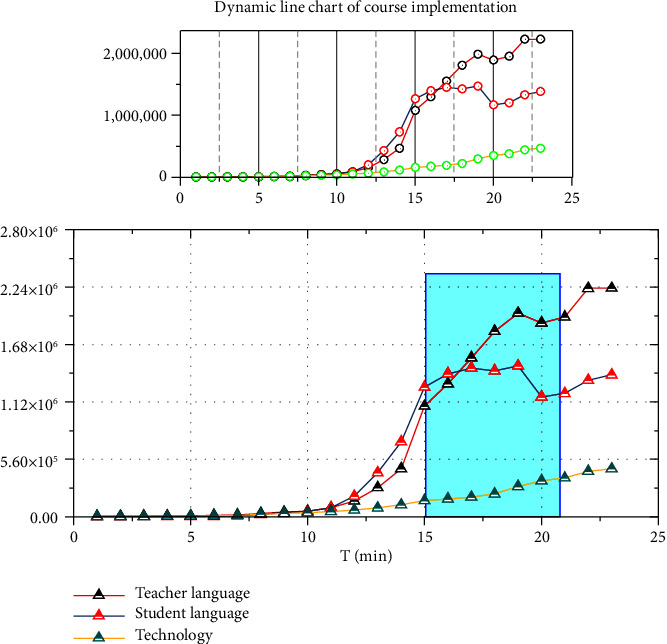
Dynamic line chart of course implementation.

As shown in Figure 5, the dynamic line graph visually shows that the teacher language and student language in the course are intertwined most of the time. The dynamic line graph no longer shows that the teacher's language is always higher than the student's language, which indicates that the teacher's interactive language is effectively controlled, that the student's speaking rate has increased, and that there is a breakthrough in the technical aspects compared to before. The results of the above data show that the implementation of the multichannel interaction model in this class was relatively smooth and achieved the expected teaching effect. The results also proved that the multichannel interactive mode can optimize the online Chinese interactive teaching, thus improving the online Chinese interactive teaching system and enhancing the interactive effect of the online Chinese classroom.

**Figure 6:**
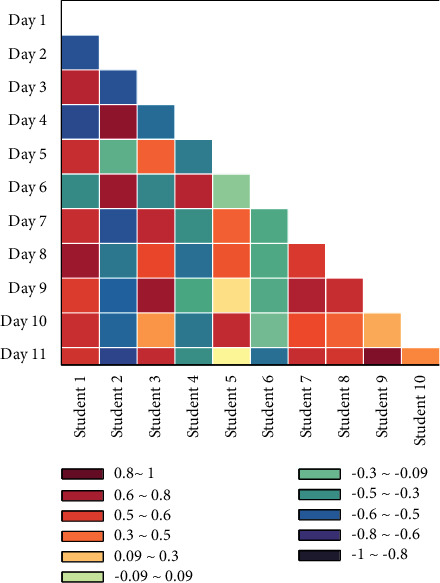
The display chart of students' learning hours.

Teachers can make microadjustments to the platform used in the process of choosing a platform for their lecture content, but overall, it should be consistent with the multichannel interactive mode. Teachers can also choose a live teaching platform in the midclass session and then choose to use a supplementary interactive teaching platform at the same time to enrich the form of interaction by using its interactive features. In addition to the choice of teaching platform, the model will have different forms and different types of interaction before, during, and after the class. For example, in the preclass recording session, the interaction is mainly delayed interaction, and the types of interaction include students' interaction with the video resource course during self-study and the predictive interaction in which the teacher tests students' self-study. In the in-class live session, the interaction is mainly real-time, and the categories of interaction include contextual interaction, game-based interaction, and topic-based interaction. Therefore, how to enhance the effect of interactive teaching in online Chinese teaching is an urgent problem to be solved in online Chinese teaching. In the postclass session, real-time interactions are using instant messenger and delayed interactions using an online learning platform, and the categories of interactions mainly include question and answer and discussion. Teachers understand students' self-learning situations through the background data. After the teacher uploads the recorded video to the platform, students conduct self-study. Teachers can monitor the length of time students watch the recorded video in the background, and the length of time students watch the video can also reflect the seriousness of students' self-study. As shown in Figure 6, the darker the green bar is, the longer the students have been watching.

In terms of design, the mobile learning resources for spoken Chinese are simple and logical, providing feedback on learners' actions. However, IH5 may have high network requirements, and the network speed will affect the learners' experience. We will further explore the aesthetics of the interface in the future. Although there were very few learners who did not know how to operate the system, they were able to master the method successfully with guidance. Therefore, the operation of the mobile learning resources for spoken Chinese is still relatively simple and easy to get started. First, it breaks the constraints of time and space; second, it can make full use of the advantages of multimedia; third, it can meet the diverse needs of students and individualized learning.

**Figure 7:**
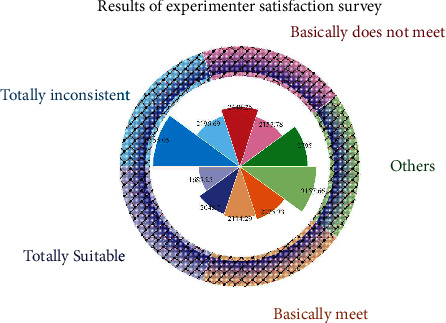
Results of the experimenter satisfaction survey.

For the survey on the experimenter's recognition of the mobile learning resource for spoken Chinese, we relied on two main factors: whether they were willing to share the learning process with their friends and whether they were willing to continue to use it for learning, and the statistical results are shown in Figure 7. The experimenters were willing to introduce the spoken Chinese mobile learning resource to their friends. This practice can help the spoken Chinese mobile learning resource expand its users and have a basis for development. At the same time, it also proves that the spoken Chinese mobile learning resources are more attractive to learners. The experimenters were willing to continue to use the mobile learning resources for spoken Chinese for learning, and they thought this method was more effective and interesting. At the same time, they are overseas and usually cannot meet many Chinese people, so distance learning can help them learn more about Chinese accents and speaking speed.

## 6. Discussion

Through questionnaires and personal interviews, many affirmations and suggestions were obtained. Most of the learners thought that mobile learning resources for spoken Chinese could meet their learning needs, and some interviewees thought that besides using film and TV resources as learning content, Chinese songs and so on could also be used as learning content; in response to the study on the fun of mobile learning resources for spoken Chinese, most of the learners said they would prefer to practice speaking in this way rather than practicing on their own against a book. The three Chinese teachers also said that using this method to practice speaking is very suitable for students to practice, especially those at the elementary level. In terms of design, the Chinese teachers thought that the pages should be simpler, keywords could be marked with other different colors, and beginners should add pinyin and translation to the video subtitles; both teachers and learners surveyed said they would be willing to use or recommend to their friends the use of mobile learning resources for spoken Chinese.

However, there are two sides to everything. When students study online, they mostly live in their own homes, and homes are living places, and the living place learning atmosphere is naturally insufficient. Therefore, students' motivation in online learning will be affected by the transfer of learning place. In online teaching, because students lack such a real classroom environment and are alone in their own learning space, some classroom activities that can be done in offline classes cannot be done online. Therefore, the limitations of the online teaching space also cause a decrease in the proportion of interaction between students. Second, the verbal communication between teachers and students throughout the classroom needs to be realized through sound transmission. The medium of sound information transmission between teachers and students, students and students has changed. In online teaching, sound becomes a signal and is transmitted through fiber optics and other substances, which will be affected by the network in the transmission process. The lag and delay of the network will affect the output of the teacher and the reception of the students; therefore, the effect of interaction will be affected and presented as less than ideal. In the process of teaching Chinese online, some Chinese teachers' classes are rich and interesting, while others' classes are limited to textbook texts and pictures. Teachers' use of resources applicable to online Chinese teaching is very important for online Chinese courses. Due to the lack of a systematic library of resources applicable to online Chinese teaching in the field of teaching Chinese as a foreign language, it takes a lot of time for teachers to prepare for their lessons.

## 7. Conclusion

With the continuous development of virtual simulation technology, the combination of remote international Chinese learning with AR, VR, 3D, and other technologies provides a new way for the development of Chinese teaching. Designing international Chinese virtual simulation mobile learning software to solve the current problems and focus on breakthroughs has a very promising application. Based on the concept of situational cognition and with the help of the A-frame framework, this paper designs task-based virtual courseware using the content of international Chinese language education, trying to explore the feasibility of combining virtual technology with international Chinese language education, and understand the advantages and problems of VR education in Chinese language teaching in the process of developing learning. The research results show that the distance international Chinese teaching model based on situational cognitive theory has a positive effect on students' Chinese learning. Learners with lower Chinese proficiency can learn again the knowledge points they have not mastered in class through the platform, and learners with higher Chinese proficiency can use the platform to consolidate the knowledge points they have learned and enrich their knowledge of Chinese culture. However, due to the epidemic, the number of Chinese learners in the selected institutions is limited, and there are limitations in the sample collection, so the quantitative analysis of the data is lacking. Future research on international Chinese language teaching should focus on contextual and applied research, improve research methods, and conduct in-depth learner-centered research on intercultural Chinese language teaching.

## Data Availability

The data used to support the findings of this study are available from the author upon request.
